# Molecular characterization of *Glaesserella parasuis* strains circulating in North American swine production systems

**DOI:** 10.1186/s12917-023-03698-x

**Published:** 2023-08-28

**Authors:** Robert Mugabi, Ana Paula S. Poeta Silva, Xiao Hu, Marcelo Gottschalk, Virginia Aragon, Nubia R. Macedo, Orhan Sahin, Perry Harms, Rodger Main, Alexander W. Tucker, Ganwu Li, Maria J. Clavijo

**Affiliations:** 1 Department of Veterinary Diagnostic and Production Animal Medicine, Iowa State University, Ames, IA USA; 2 Groupe de Recherche Sur Les Maladies Infectieuses du Porc, Faculté de Médecine Vétérinaire, Université de Montréal, Montréal, Canada; 3 Centre de Recerca en Sanitat Animal (CReSA), Unitat Mixta d’Investigació IRTA-UAB en Sanitat Animal, UniversitatAutònoma de Barcelona (UAB), Campus, Bellaterra, Barcelona, Spain; 4 Programa de Sanitat Animal, Centre de Recerca en Sanitat Animal (CReSA), IRTA, UniversitatAutònoma de Barcelona (UAB), Campus, Bellaterra, Barcelona, Spain; 5 WOAH Collaborating Centre for the Research and Control of Emerging and Re-Emerging Swine Diseases in Europe (IRTA-CReSA), Bellaterra, Barcelona, Spain; 6 PIC North America, Hendersonville, TN USA; 7 Department of Veterinary Medicine, University of Cambridge, Cambridge, CB3 0ES UK

**Keywords:** *Glaesserella parasuis*, Swine, Polyserositis, Serotyping, Whole-genome sequencing, Multilocus sequence typing, Phylogeny

## Abstract

**Background:**

*Glaesserella parasuis* is the causative agent of Glässer’s disease in pigs. Serotyping is the most common method used to type *G. parasuis* isolates. However, the high number of non-typables (NT) and low discriminatory power make serotyping problematic. In this study, 218 field clinical isolates and 15 *G. parasuis* reference strains were whole-genome sequenced (WGS). Multilocus sequence types (MLST), serotypes, core-genome phylogeny, antimicrobial resistance (AMR) genes, and putative virulence gene information was extracted.

**Results:**

In silico WGS serotyping identified 11 of 15 serotypes. The most frequently detected serotypes were 7, 13, 4, and 2. MLST identified 72 sequence types (STs), of which 66 were novel. The most predominant ST was ST454. Core-genome phylogeny depicted 3 primary lineages (LI, LII, and LIII), with LIIIA sublineage isolates lacking all vtaA genes, based on the structure of the phylogenetic tree and the number of virulence genes. At least one group 1 *vtaA* virulence genes were observed in most isolates (97.2%), except for serotype 8 (ST299 and ST406), 15 (ST408 and ST552) and NT (ST448). A few group 1 *vtaA* genes were significantly associated with certain serotypes or STs. The putative virulence gene *lsgB*, was detected in 8.3% of the isolates which were predominantly of serotype 5/12. While most isolates carried the *bcr*, *ksgA*, and *bacA* genes, the following antimicrobial resistant genes were detected in lower frequency;  *blaZ* (6.9%), *tetM* (3.7%), *spc* (3.7%), *tetB* (2.8%), *bla-ROB-1* (1.8%), *ermA* (1.8%), *strA* (1.4%), *qnrB* (0.5%), and *aph3''Ia* (0.5%).

**Conclusion:**

This study showed the use of WGS to type *G. parasuis* isolates and can be considered an alternative to the more labor-intensive and traditional serotyping and standard MLST. Core-genome phylogeny provided the best strain discrimination. These findings will lead to a better understanding of the molecular epidemiology and virulence in *G. parasuis* that can be applied to the future development of diagnostic tools, autogenous vaccines, evaluation of antibiotic use, prevention, and disease control.

**Supplementary Information:**

The online version contains supplementary material available at 10.1186/s12917-023-03698-x.

## Background

*Glaesserella parasuis* (*G. parasuis*) is a Gram-negative bacterium that causes Glässer’s disease in pigs. This disease is characterized by arthritis, meningitis, and polyserositis, commonly observed in 4 to 8-week-old pigs, but it can also sporadically occur in older pigs [1]. Clinical signs are abdominal breathing or coughing, lameness, paddling, and septicemia with acute death [2, 3]. While the agent was first described in 1910 [1], it continues to challenge the health and productivity of swine production systems today. In fact, over the last ten years, disease due to *G. parasuis* increased substantially in swine cases received at the Iowa State University Veterinary Diagnostic Laboratory (ISU-VDL) [4].

The bacterium is considered endemic in all swine populations, colonizing the upper respiratory tract of pigs. Since the pathogenicity of *G. parasuis* strains varies quite significantly, from highly pathogenic, such as the well-described Nagasaki strain (serotype 5 ST24), to non-pathogenic strains, such as strains of serotype 3, 6, 9, and 11 [5, 6], effective control hinges upon the correct identification and typing of the disease-causing strains. Serotyping has been the most commonly used typing method, with 15 serotypes based on capsular polysaccharides described to date [5, 7]. The major pitfall of serotyping is its low discriminatory power and typeabilty. Multilocus sequence typing (MLST) [8, 9] is an alternative typing technique. Although MLST provides better strain discrimination and portability compared to serotyping, it is labor-intensive and time-consuming, hindering adoption. A plethora of other GPS genotyping methods have been described previously for instance pulsed field gel electrophoresis (PFGE), amplified fragment length polymorphisms (AFLP), multilocus variable number of tandem repeat analysis (MLVA), random amplified polymorphic (RAPD), enterobacterial repetitive insertion consensus PCR (ERIC-PCR) [10–15]. Some of these methods have been compared previously [16]. Although most of these methods have relatively good discriminatory power, the results are hard to compare between laboratories, interpretation of the banding pattern can be ambiguous, and do not in general provide information on the virulence potential of a given strain.

Pathotyping is also available for *G. parasuis.* The most important virulence factors described for *G. parasuis* are the virulence-associated trimeric autotransporter (*vtaA*) genes [6]. There is increasing evidence that group 1 *vtaA* genes are associated with virulent isolates [17, 18]. While other putative virulence-associated genes, [19–22] have been described, these genes are not targeted frequently in diagnostic settings to assess the virulence potential of strains.

The reduction in sequencing costs and advances in bioinformatic pipelines have made whole genome sequencing (WGS) more feasible for diagnostic use. WGS offers higher discrimination between isolates by allowing the extraction of relevant information related to phylogeny, sequence type, putative virulence factors, and antimicrobial resistance (AMR) genes. Current knowledge about the molecular epidemiology of disease-associated *G. parasuis* strains circulating in North American pig populations is lacking. The objective of this study was to perform genetic characterization of *G. parasuis* strains from disease-associated cases using WGS. In-depth characterization using WGS could vastly improve on farm control and prevention programs.

## Results

### Phylogenetic analysis

**Fig. 1:**
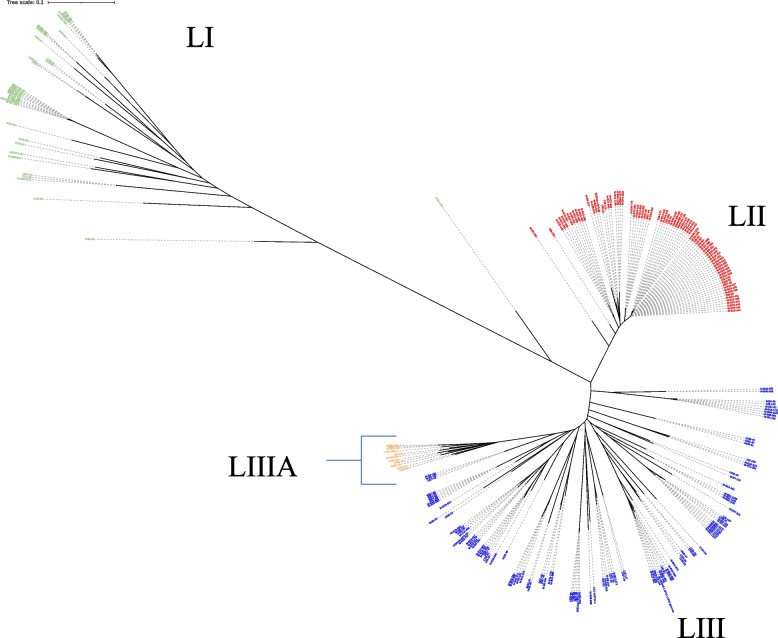
Core-genome phylogenetic tree of 218 sequenced genomes and 15 reference strains of *G. parasuis* isolates. A phylogenetic tree was constructed based on core-genome SNPs. Color shades indicate the primary lineages; LI, LII, and LIII. One isolate between LI and LII is an outlier

**Fig. 2:**
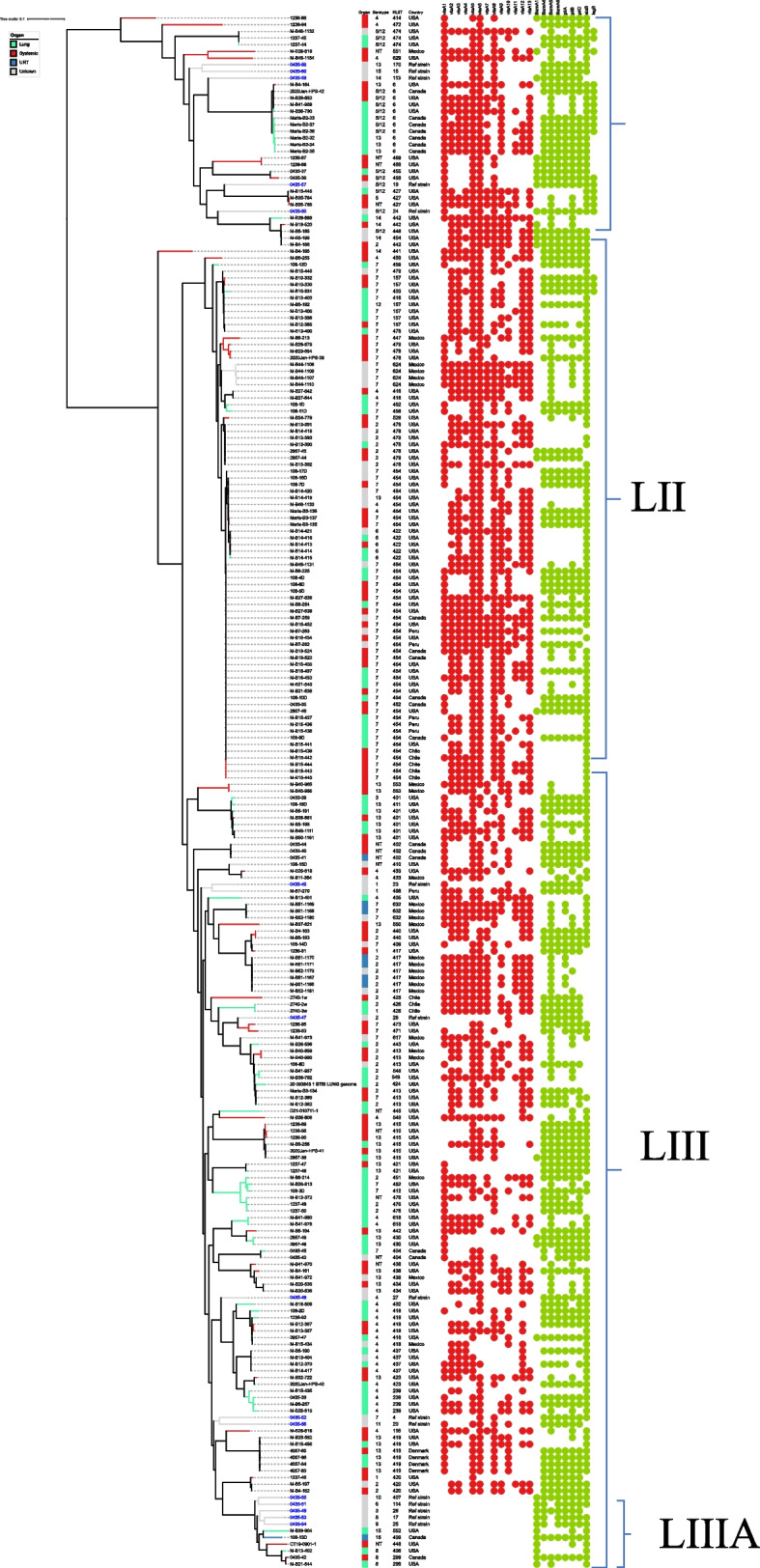
Distribution of selected virulence-associated genes. The phylogenetic tree was constructed based on core-genome SNPs. The red and green dots indicate the relative distribution of the *vtaA* genes and other putative virulence genes

At least 47,061,190 of the paired-end reads processed had a Phred score of 30, suggestive of high quality. The overall GC content was between 39.06 and 40.26. A total of 71,368 core genome SNPs were identified from all strains by kSNP3. The phylogenetic tree based on the core-genome SNPs of these isolates indicated high genetic diversity, and were clustered into three primary lineages (LI, LII, LIII) (threshold = 0.1) based on the structure of the phylogenetic tree and the number of virulence genes (Fig. 1). The largest primary lineage was LIII, containing 49.5% (*n* = 108) of the study isolates. LI and LII contained 13.8% (*n* = 30) and 36.2% (*n* = 79) of the isolates, respectively. Each lineage contained sub-lineages with only LIIIA sublineage being highlighted due to its unique characteristics (Fig. 2).

### Serotype, sequence type, and clonal complex

**Fig. 3:**
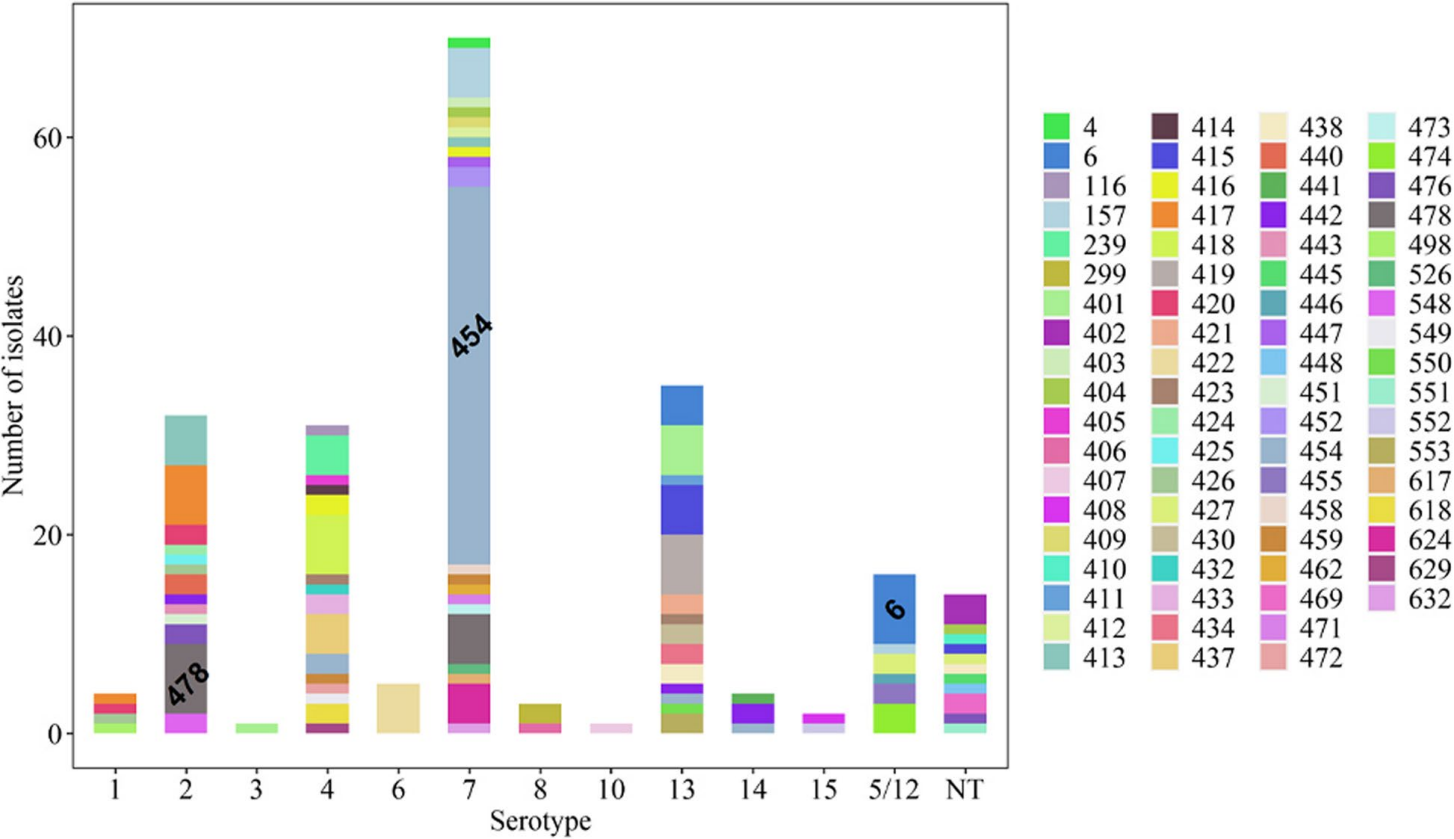
Distribution of sequence types (STs) and serotypes of *Glaesserella parasuis* (*G. parasuis*) isolates. The colour codes represent the sequence type diversity within in a given serotype. Frequently detected STs ST454, ST478, and ST6 are highlighted

Among field isolates (*n* = 218), in silico whole-genome serotyping pipeline identified 11 of the 15 known *G. parasuis* serotypes (Fig. 3) with serotypes 9, 10, and 11 not being detected. A total of 6.4% (14/218) of isolates were untypable. The Simpson’s index of diversity for serotyping was estimated as 0.82. The most frequently detected serotypes were 7 (32.6%, 71/218), 13 (16.1%, 35/218), 2 (14.7%, 32/218), 4 (14.2%, 31/218), and 5/12 (7.3%, 16/218). Each lineage had unique predominant serotype (s). Within LI, LII, and LIII isolates, serotype 5/12 (50%, 15/30), 7 (75.9%, 60/79), and 13 (27.8%, 30/108), were the most frequently detected serotypes, respectively (Table 1). Consistent with the above distribution of serotypes within lineages, statistical analysis showed that serotype 5/12 was associated with LI, serotype 7 was associated with LII, and serotypes 2, 4, and 13 were associated with LIII (*p* < 0.05). In addition, LI and LII isolates were significantly associated with respiratory and systemic isolates, respectively (*p* < 0.05). A total of 80 isolates were isolated from tissues for which histopathological data was available. Of these, 8 did not have detectable lesions and were of serotypes 4, 7 and 13 with majority of pigs having lesions in other sites in which *Streptococcus suis* isolated. Among the 72 remaining isolates, the following lesions and serotypes were detected: polyserositis (*n* = 27) bronchopneumonia/pneumonia (*n* = 26) epicarditis/myocarditis (*n* = 9); meningitis/encephalitis (*n* = 3) arthritis/synovitis (*n* = 4), pleuritis (*n* = 2) splenitis (*n* = 1) (Additional file 3).

**Table 1:** Characteristics of isolates in the specific phylogenetic lineages

Lineage	Number of isolates	Number of flows	Country (n)	Serotype (%) ^b^	ST	Average number of group 1 *vtaAs*	Putative virulence factors (%)^b^	AMR genes (%)^b^
I	30	12	Canada (*n* = 7), Mexico (*n* = 1) and USA (*n* = 22)	2 (3.3%) 4 (10%) 13 (13.3%) 14(10%) 5/12 (50%) NT (13.3%)	6, 414, 427, 442, 446, 454, 455, 469, 472, 474, 551, 629	6.8	*bmaA4*, *bmaA6*, *pilA*, *pilC* (63.3%), *bmaA1* (33.3%), *bmaA*5, *pilB* (96.7%), *lsgB* (50%), *siaB* (93.3%), *omP2* (66.7%), *omP5* (100%)	*bcr*, *ksgA*, sul2 (100%), *aph(3'')-Ib*, *bacA* (99.1%), *blaZ* (13.3%), spc (13.3%, *ermA* and *tetB* (3.3%), *tetM* (6.7%)
II	79	25	Canada (*n* = 6), Chile (*n* = 6), Mexico (*n* = 5), Peru (*n* = 5), USA (*n* = 57)	2 (8.7%) 4 (6.3%) 6 (6.3%) 7 (75.9%) 5/12 (1.3%) 13 (1.3%)	157, 403, 416, 422, 447, 452, 454, 458, 459, 462, 478, 526, 624	6.2	*bmaA1*, *lsgB* (3.8%), *bmaA4, bmaA6, pilA, pilB, pilC* (40.5%), *bmaA5* (62%), *omP2* (53.2%), *omP5* (100%), *siaB* (93.7.1%)	*bcr*, *aph (3'')-Ib*, *ksgA*, *sul2* (100%), *bacA* (98.7%)
III	108	40	Canada (*n* = 7), Chile (*n* = 3) Denmark (*n* = 4), Mexico (*n* = 19), Peru (*n* = 1) USA (*n* = 74)	1 (3.7%) 2 (22.2%) 3 (0.9%) 4 (21.3%) 7 (10.2%) 8 (2.7%) 13 (27.8%) 15 (1.9%) NT (9.3%)	116, 239, 299, 401, 402, 404, 406, 408–413, 415, 417–421, 423, 424–426, 430, 432–434, 437, 438, 440, 442,443,445, 448, 451, 452, 471, 473, 476, 498, 548, 549, 552, 553, 617, 618, 632	5.02	*bmaA1* (9.3%), *omP2*, *pilC* (56.5%), *bmaA4 bmaA6* (55.6%), *bmaA5* (85.2%), *omP5* (100%), *pilB* (72.2%), *siaB* (75%), *pilA* (62%)	*bcr*, *aph(3'')-Ib*, *ksgA*, *sul2* (100%), *bacA* (98.1%), *qnrB*, *aph3''Ia* (0.9%), *bla *_*ROB-1*_, *spc* (3.7%), *strA*, *ermA* (2.8%), *tetJ* (2.7%, *tetM* (5.6%), *tetB* (4.6%)

^b^Represents percentage of a given serotype, putative virulence factor, or AMR gene within a lineage

Through multilocus sequence typing analysis, we uncovered a total of 72 sequence types (STs) within the collection of 218 field isolates. Remarkably, among these, 66 STs were entirely new discoveries, representing 91.7% of the total STs identified, with ST454 emerging as the most commonly encountered one (Fig. 3, Additional file 3). The Simpson’s index of diversity (SDI) for MLST was 0.95. All reference strains typed as previously known STs, except serotype 10 (Additional file 3). In contrast with serotyping data, the majority of the STs clustered within the same lineage based on whole-genome SNP phylogenetic analysis. The majority of isolates belonged to a limited number of clonal complexes (CCs), which included CC157 (24.7%, *n* = 54), CC478 (8.3%, *n* = 18), CC413 (4.6%, *n* = 10), CC417 (3.7%, *n* = 8), CC56 (2.8%, *n* = 6), CC92 (2.8%, *n* = 6), CC452 (2.8%, *n* = 6), CC442 (2.3%, *n* = 5), CC438 (1.4%, *n* = 3), CC245 (0.9%, *n* = 2),), CC96 (0.9%, *n* = 2), CC433 (0.9%, *n* = 2), and CC246 (0.5%, *n* = 1). The most common CCs were CC157 and CC478 with CC157 comprising of STs, ST416, ST454, ST458, ST462, ST526, and ST157. CC478 was the second most frequently detected clone containing ST459, ST478, and ST624 (Additional file 1). ST478 is a single locus variant (SLV) of ST157 but formed its own clone (Additional file 1). Based on the group definition criterion, 43.4% (95/218) of the STs were considered singletons. ST6, ST454, and ST478 were associated with serotypes 5/12, 7, and 2, respectively (*P* < 0.05). However, ST6 strains were also from serotypes 13, ST454 of serotypes 4, 13 and 14, and ST478 of serotypes 7. A high ST diversity within serotypes was observed. For example, within serotype 7 strains, a total of 19 different STs were identified. Similar findings were observed with serotype 13 and 2. The untypable strains were of different sequence types.

Within the dataset, the three dominant STs were ST454 (19.3%, 42/218), ST478 (5.5%, 12/218), and ST6 (5%, 11/218). The remaining STs were each represented by ≤ 7 isolates, and 33/72 STs were represented by a single isolate. The two novel STs (454 and 478) are single locus variants (SLVs) of each other at the *infB* locus. Within LI and LII, ST6 (36.7%, 11/30) and ST454 (51.9%, 41/79) were the dominant STs, respectively, while LIII showed high diversity with 49 different STs. All six isolates in the LIII A sublineage (Fig. 2) were identified as novel STs, except for two serotype 8 isolates that were both ST299. ST6 was associated with LI (*p* < 0.05), and ST454 and ST478 were significantly associated with LII (*p* < 0.05). From cases with histopathology data, most ST454 and ST478 isolates originated from polyserositis cases.

### Distribution of virulence-associated genes

A total of 212 (97.2%) isolates possessed at least one of the group 1 *vtaA* genes. Within group 1 *vtaA* genes*, vtaA5* (88.1%, 192/218), *vtaA6* (84.7%,185/218), *vtaA3* (67.4%, 147/218), *vtaA8* (66.5%, 145/218), and *vtaA2* (65.1%, 142/218) were the most frequently detected (Additional file 3). The remaining group 1 *vtaA* genes were *vtaA1*, *vtaA9*, *vtaA4* and *vtaA*7, with 58.7% (128/218), 53.2% (116/218), 43.1% (94/218) and 30.7% (67/218) presence, respectively. On average, LI isolates carried 6.8 (min: 2, max: 9) group 1 *vtaA* genes, LII isolates carried on average 6.2 (min: 4 max: 9) group 1 *vtaA* genes, and LIII isolates carried an average 5.02 (min: 1, max: 9) group 1 *vtaA* genes, excluding LIIIA. Sublineage LIIIA isolates lacked all *vtaA* genes (Fig. 2). *VtaA3*, *vtaA8*, and *vtaA9* were significantly associated with LI and LII (*p* < 0.05). *VtaA1*, *vtaA4*, and *vtaA7* were significantly associated with LI, and *vtaA6* was associated with LIII.

**Fig. 4:**
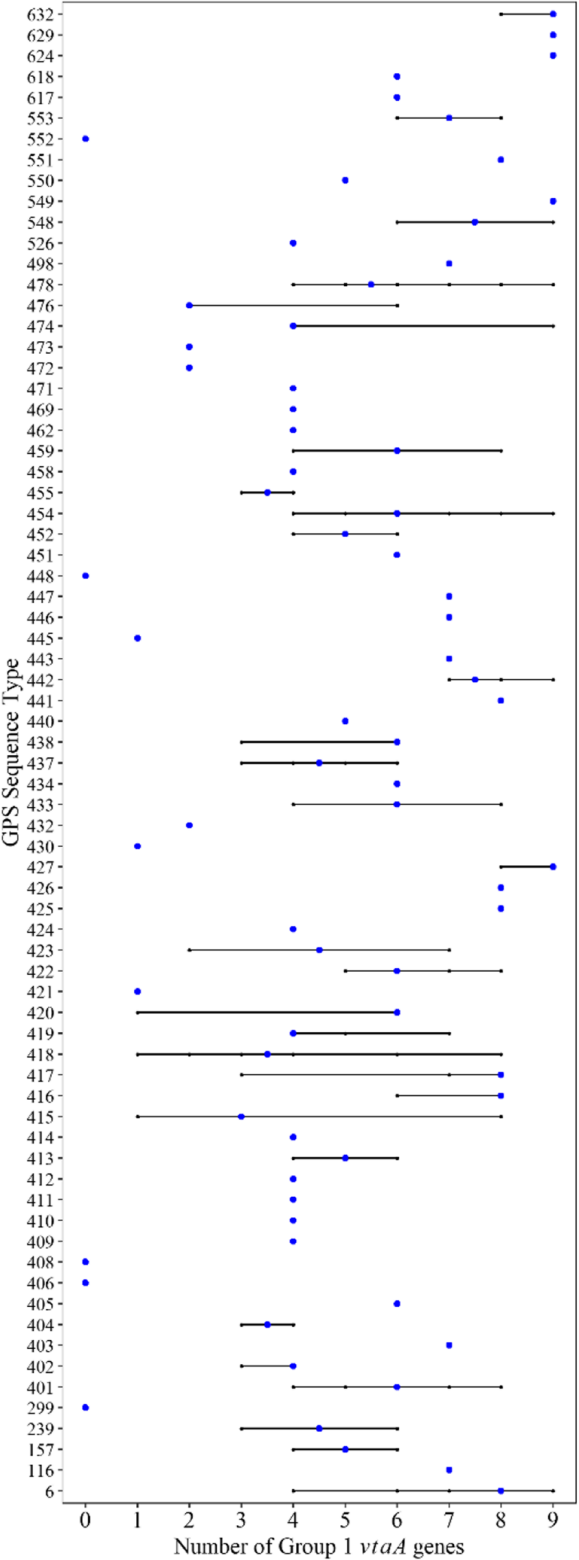
Number of Group 1 *vtaA* genes by ST. Black line represents the range of Group 1 *vtaA* genes with blue dot identifying the median

A total of 23 isolates, predominantly within LI and LII, carried all 9 group 1 *vtaA* genes and were of serotypes 7 ST454, ST624, and ST632, serotype 5/12 ST6, ST427, and ST474, serotype 2 ST478 and ST548, serotype 4 ST549 and ST629, serotype 13 ST6, serotype 14 ST442, and NT ST427. From these 23 isolates, six also carried all the group 2 and 3 *vtaA* genes, and were of serotype 7 ST454 and ST624 and serotype 14 ST442. Some isolates represented by the same ST, carried a varied number of group 1 *vtaA* genes (Fig. 4).

Group 2 *vtaA* genes were found in the following proportions: *vtaA10* (34.4%, 74/218) and *vtaA11* (23.4% 51/218). Finally, within group 3 *vtaA* genes, *vtaA12* was found in 56.4% (123/218) and *vtaA13* in 61.5% of isolates, respectively (134/218).

Within serotypes, *vtaA1* was associated with serotype 5/12, *vtaA2* and *vtaA7* were associated with serotype 2, and *vtaA5*, *vtaA6* and *vtaA8* were associated with serotype 7 (*p* < 0.05). Among the dominant STs, *vtaA1*, *vtaA4*, *vtaA7*, and *vtaA9* were associated with ST6, and *vtaA8 and vtaA9* were associated with ST454 (*p* < 0.05).

The prevalence of other putative virulence genes was further determined using whole-genome sequencing analysis. All isolates carried the porin protein *ompP5* gene, and 56.9% (124/218) of the isolates were also positive for the porin protein gene *ompP2*. Fimbrial genes (*pilA*, *pilB*, and *pilC*) and *siaB* were present in 54.6% (119/218), 69.3% (151/218), 51.8% (113/218), and 84.4% (184/218) isolates, respectively. Among the monomeric autotransporter genes, *bmaA5* (78.4%, 171/218), *bmaA4* (51.3%,112/218)*,* and *bmaA6* (51.3% (112/218), were the most prevalent while bmaA*1* (10.6%, 23/218) was the least prevalent. The *lsgB* gene was the least prevalent gene among the selected putative virulence genes, with a proportion of only 8.3% (18/218) of isolates being positive; all isolates in LIII lacked this gene. Almost all *lsgB* positive isolates (15/18) were part of L1 (Table 1), and were predominantly serotype 5/12.

### Identification of antimicrobial resistance genes

Fourteen AMR genes were detected among the 218 genomes, encoding resistance to antibiotics of nine different classes including 2,5-diketopiperazines, aminoglycosides, beta-lactams, liconsamides, macrolides, lincosamide streptogramins B (MLS), polypeptides, quinolones, sulfonamides, and tetracyclines. Among these, *bcr*, *ksgA,* *bacA,* sul2, and *aph (3'')-Ib* were detected in almost all isolates included in the collection. The remaining genes were present at the following proportions; *blaZ* (6.9%, 15/218, *tetM* (3.7%, 8/218), *spc* (3.7%, 8/218), *tetB* (2.8%, 6/218), *bla-*_ROB-1_ (1.8%, 4/218), *ermA* (1.8%, 4/218), *strA* (1.4%, 3/218)), *qnrB* (0.5%, 1/218), and *aph3''Ia* (0.5%, 1/215).

### Relationships between strains and geographical location, tissue, and disease

The distribution of *G. parasuis* isolates was further analyzed geographically. A total of 76% of isolates (19/25) from Mexico, 48% from the USA (74/154), and 35% from Canada (7/20) were grouped into LIII; 20% (5/25), 37% (57/154), and 30% (6/20) of the isolates from Mexico, USA, and Canada, respectively, clustered in LII. Finally, 4% (1/25), 14.2% (22/154), and 35% (7/20) of Mexico, the USA, and Canada isolates, respectively, were grouped into LI. A few isolates from Chile, Peru, and Denmark clustered in specific lineages (Additional file 3). Lung (*n* = 86) and systemic strains (*n* = 89) accounted for 80.3% of the isolates. The remaining were from URT (*n* = 8) or unknown (*n* = 35). Generally, there was no observed tissue-specific distribution within the lineages. In several isolates (*n* = 80) from tissues with reported histopathological lesions, serotypes associated with disease were 7, 13, 4, 5/12 and 2.

## Discussion

*G. parasuis* continues to challenge the productivity, health, and well-being of post-weaning pig populations, and represents a driver of antimicrobial use on farms [1]. Effective control of *G. parasuis* requires a multifaceted approach based on the judicious use of antimicrobials, vaccination, minimizing viral co-infections and environmental triggers, improving husbandry and characterization of the disease-causing strains. Whole-genome sequencing analysis provides multiple layers of information that aid in determining the clinical relevance of the isolates and providing epidemiological insight.

In this study, WGS was performed on 218 *G. parasuis* isolates predominantly from North America. The size and GC content were within range with other published *G. Parasuis* genomes [23]. Phylogenetic analysis revealed three primary lineages, LI, LII and LIII. This is in contrast with a recently published study from China [23] that showed *G. parasuis* isolates clustering into two main primary lineages comprised of STs not found in the present study, highlighting the high genetic variation found within the species.

Whole-genome in silico serotyping revealed 11 of the 15 known serotypes, with serotypes 7, 13, 4, 2, and 5/12, as the most common serotypes detected. The WGS in silico serotyping scheme was based on the DNA sequence fragment for each serotype published in Howell et al.[24]. The scheme is unable to discriminate serotype 5 and 12. However, future comparative genomic analysis studies using complete genomes could be used to determine whether these two serotypes can be discriminated. In this study, only 6.4% of the isolates were untypable via in silico serotyping. This percentage was lower compared to 39% and 9.7% that utilized traditional and PCR based serotyping methods [10, 25], respectively, emphasizing the potential advantage of sequence-based methods for bacterial typing. Furthermore, a real-time PCR serotyping scheme recently developed was able to assign a serotype to all 40 isolates that were previously untypable by conventional serotyping [26].

The most frequently detected serotypes observed in this study have been previously associated with disease and are commonly detected from clinical cases [10, 25, 27, 28]. However, in most studies serotypes 4 and 5/12 were the most frequently detected [10, 29–31]. This study further supports the increasing evidence for serotype 7 in disease [25, 31]. In a recent study, serotype 7 strains from clinical cases were frequently detected in USA, Canada, Europe, China, and Vietnam, and some were associated with virulence [25]. Its widespread nature could be attributed to trade between these countries or companies moving pigs between them.

Multilocus sequence typing identified 72 sequence types within 218 isolates, of which 91.7% (66/72) were novel. Specifically, this study highlighted the emergence of ST454, a strain detected in outbreaks of high mortality, characterized by per acute disease and polyserositis. The *eBurs*t analysis showed that ST454 belonged to the CC157 with its predicted founder ST157, a sequence type first identified in Brazil from a pig with bronchopneumonia (https://pubmlst.org/organisms/glaesserella-parasuis). Surprisingly, ST157 was among the least prevalent STs in this study. In contrast, a clonal complex’s ancestral ST (founder) is typically the most prevalent ST in a population due to the fitness advantage or random genetic drift [32]. It is likely that as the number of isolates in the database increases, the founder ST might change to ST454.

The *eBurst* analysis revealed that almost half of the isolates had singleton STs, suggesting high heterogeneity and instability in the population structure, as previously described [11, 17, 33]. The ST diversity observed in this study has also been reported in other studies. Olvera et al*.* identified 122 STs within 150 strains (17), Turni et al*.,* identified 54 STs (41 novel) within 75 isolates from Australia [11]. In this study, only 5 of the 72 STs identified had been previously reported, further highlighting the significant heterogeneity of the species on a global scale. While a high diversity of STs was observed, only a few STs, including ST454, ST478, and ST6 were the most frequently detected in the current study. The high detection frequency of these STs could reflect unique production systems submitting samples to the diagnostic laboratory and not a true reflection of the overall prevalence. Still, the data shows the clear role of these STs in disease cases in the North America. U.S, but also their distribution in different countries and flows. For example, ST454 was detected in 24 distinct flows and 5 countries. Targeted control and elimination procedures, such as inclusion of these STs in autogenous vaccine products or depopulation of herds carrying such STs could potentially reduce the *G. parasuis* disease burden for a significant number of production systems. While the intensity of sampling varied across production systems, multiple flows had at least 2 STs (Additional file 2), supporting previous knowledge that within a production system multiple *G. parasuis* strains can be detected [34] and could be contributing to disease. Therefore, disease control should rely on the characterization of the clinically relevant strains within a flow to determine the appropriate vaccine candidates for autogenous vaccine production or to evaluate the potential efficacy of commercially available vaccines, as well as to improve the sourcing of replacement animals and pig flow management. In this study, while multiple serotypes and STs were detected within most flows, several flows were represented by a predominant ST or serotype (Additional file 1), particularly genotypes with apparent higher pathogenicity, such as with ST454.

Unlike serotypes, isolates of the same STs were mostly lineage specific, as revealed in the WGS-based phylogenetic analysis (Fig. 2). Furthermore, isolates of the same serotypes had multiple different STs but the opposite was observed less frequently, highlighting the higher discriminatory power of MLST compared to serotyping. Furthermore, core-genome SNP phylogenetic analysis showed evidence of genetic variation in isolates within the same STs e.g. ST6 and ST454 (Fig. 2), demonstrating the higher resolution offered by whole-genome sequencing compared to MLST and serotyping. Still, whether these genomic differences between strains of the same STs impact the clinical outcome is yet to be elucidated.

Among the virulence-associated genes, group 1 *vtaA* genes are good predictors of virulence, and some have been shown experimentally to play a role in disease [17, 18, 35, 36]. Approximately 97% of isolates from this study carried at least 1 group 1 *vtaA* gene. Based on previous research [25, 36], this suggests that these strains could have pathogenic potential. Phylogenetic SNP analysis revealed three primary lineages with varied number of average group 1 *vtaA* genes present. LI and LII isolates carried a numerically higher number of group 1 *vtaA* genes on average than LIII, whereas isolates from the LIIIA sublineage (Fig. 2) lacked all the *vtaA* genes. It is not entirely known if there is a correlation between the number of group 1 *vtaA* genes and increased virulence or if certain patterns of *vtaA* gene carriage can be a predictor of virulence. However, we observed some group 1 *vtaA* genes were significantly associated with known serotypes associated with disease for example 5/12 or 7, suggesting that these could serve as predictors of virulence. Isolates of serotype 8 and 15 in LIIIA lacked all *vtaA* genes and are serotypes that have been predominantly detected from the nasal cavity of pigs, confirming previous findings [25]. Variability in carriage of group 1 *vtaA* genes was noticed within isolates of the same ST or genetically similar based on SNP phylogenetic analysis (Fig. 2). For instance, group 1 *vtaA* gene carriage for 39 serotype 7 ST454 isolates ranged from 4–9. Similarly, all CC157 isolates (*n* = 54) carried between 4 and 9 group 1 *vtaA* genes. This shows how variable *vtaA* carriage can be even between strains showing high similarity at the core -genome level. The lack of detection of group 3 *vtaA* genes 12 and 13 in 44% and 33% of isolates, respectively, was an unexpected finding since previous studies have identified these genes among all *G. parasuis* strains previously examined but this was based on the translocator domain PCR (17). Some *VtaA12* and *vtaA13* might be divergent and therefore not identified as *vtaA12* or *vtaA13* homologs. In this study, genes were only reported if their sequence identity was above 70% and the gene coverage was above 50%. Previous studies have shown divergence within these genes which could have contributed to a lack of detection given our stringent definition for gene reporting (V. Aragon, unpublished data).

Monomeric autotransporters, porin proteins, and fimbria are involved in *G. parasuis* pathogenesis [21, 37, 38]. At least 51% of the isolates were positive for *bmaA4*, *bmaA5*, *bmaA6*, *ompP2*, *ompP5*, *pilA*, *pilB*, *pilC* and *siaB* in the current study. In fact, even isolates that were negative for all *vtaA* genes were positive for some of these, such as *ompP5 and siaB*. This may suggest that some of these putative virulence genes are conserved in both virulent and nonvirulent strains and may not be good predictors of virulence compared to group 1 *vtaA* genes. However, this notion should be interpreted with caution since the study isolates were predominantly from clinical cases. *lsgB*, which has been associated with virulent strains [20], was detected in only 8.3% of the isolates which were predominantly of serotype 5/12, suggesting a potential marker for this serotype.

Control and prevention of Glässer’s disease often rely on the strategic use of antimicrobials. Thus, detection of antimicrobial resistance genes could provide value in antimicrobial use decision-making. Among the genes, *bcr* (bicyclomycin resistance*)*, *ksgA* (kasugamycin resistance), *bacA* (bacitracin resistance*)*, *sul2* (sulfonamide resistance, and *aph (3’’)-Ib* (streptomycin resistance) were detected in almost 100% of the isolates. Consistent with this study, *bcr*, *ksgA*, *and bacA* genes were highly prevalent in *G. parasuis* isolates in a recent study [23]. Some of these highly prevalent genes encode resistance to antibiotics not commonly used in swine production. A recent study showed that tetracyclines, lincosamides, and beta-lactams are among the top three antibiotic classes used in the USA [39]. Furthermore, susceptibility data from Iowa State University shows most 2021 *G. parasuis* strains (*n* = 908) as mostly susceptible to ampicillin, ceftiofur, enrofloxacin, florfenicol, tiamulin and tilmicosin (https://vetmed.iastate.edu/sites/default/files/VDL/pdf/Susceptibility-Summary-Porcine-2021.pdf). The remaining genes; *aph3’’Ia, strA, *^*bla*^*ROB-1, blaZ*, *spc*, *ermA*, *tetB*, *tetM* and *qnrB* were detected in lower frequencies. Some studies showed varying frequencies of these genes in *G. parasuis* [40, 41]. Due to the low prevalence of some of these AMR genes a definitive conclusion on their distribution in phylogenetic lineages could not be determined. However, they were detected more frequently in LIII than LI and LII isolates (Table 1). For instance, 73.3% (11/15) and 75% (6/8) of the isolates positive for *blaZ* and *tetM*, respectively, were in LIII, and were of serotypes 1 ST417 and ST420, 2 ST476, 4 ST418, 7 ST471 and ST473, 13 ST415 and ST421, and NT ST415. Further studies are needed to correlate if the genotypic antimicrobial resistance profile can predict phenotypic resistance in this species.

Although the findings of this study were consistent with the published literature to date, caution is needed when interpreting the results. This is because the *G. parasuis* isolates included in the current study were from disease diagnosis cases submitted to the ISU VDL, and thus only captured production systems that submit samples to this laboratory. However, the contributing production systems represented the top pork producers in North America.

## Conclusion

While WGS is computational demanding, requires high technical skill and is currently more expensive than other typing tools, the value obtained with the information far exceeds these limitations. Still, it is expected that serotyping and virulence marker PCRs will continue to be used to monitor *G. parasuis* strain variation. Results herein will lead to a better understanding of the molecular epidemiology and virulence potential in *G. parasuis* that can be applied to the development of improved diagnostic tools, evaluation of antibiotic use, tracking of outbreak strains and identification of vaccine candidates for improved management of Glässer’s disease in swine production systems.

## Methods

### Source of isolates

A total of 218 *G. parasuis* isolates were obtained from porcine cases submitted to the Iowa State University Veterinary Diagnostic Laboratory (ISU VDL) from 2015 through 2022 (Additional file 3). While strains mostly originated from the USA (*n* = 154), they were also obtained from Canada (*n* = 20), Chile (*n* = 9), Denmark (*n* = 4), Peru (*n* = 6), and Mexico (*n* = 25). (Additional file 3). For each isolate, metadata was obtained, including; the year of isolation, tissue type, flow and farm name, and pig age. Strains from the USA originated from at least 16 different states. Histopathological data was obtained for 80/218 isolates (Additional file 3).

In addition, a total of 15 reference strains were provided by Dr. Marcelo Gottschalk, each representing one of the 15 *G. parasuis* serotypes [5]. All presumptive *G. parasuis* strains isolated at the ISU VDL or submitted as pure cultures from VDL clients were subjected to matrix-assisted laser desorption ionization-time-of flight mass spectrometry (MALDI-TOF MS) for definitive species identification prior to sequencing.

### Whole genome sequencing

Initial isolation of *G. parasuis* from samples was achieved by plating clinical samples onto 5% sheep blood (Hardy Diagnostics, Santa Maria, CA) with *Staphylococcus hyicus* nurse streak and incubating overnight at 37^0^C. Presumptive *G. parasuis* isolates were subcultured onto chocolate agar (Thermo scientific™), confirmed using MALDI-TOF MS, and frozen at -80^0^C in brain heart infusion broth with 30% glycerol. Two days before submission for sequencing, bacterial strains were retrieved from the freezer and streaked onto chocolate agar. An overnight pure growth culture lawn was inoculated into 2 mL phosphate-buffered saline (PBS). Genomic DNA was extracted using ChargeSwitch gDNA mini bacterial kit (Life Technologies Carlsbad, CA) according to the manufacturer’s instructions [42].

The DNA quality (A280/A260) was assessed using a Nanodrop (Thermo scientific™) and quantified using a Qubit fluorometer dsDNA HS kit (Life Technologies). Multiplex genome libraries were prepared using the Nextera XT DNA library preparation kit (Illumina, San Diego, CA). The genomic library was quantified using a Qubit fluorometer dsDNA HS kit (Life Technologies Carlsbad, CA) and normalized to the recommended amplification concentrations. The pooled libraries were sequenced on an Illumina Miseq sequencer using Miseq Reagent V3 for 600 cycles (Illumina, San Diego, CA). Raw reads were demultiplexed automatically on the Miseq.

Raw sequence reads were pre-processed using Trimmomatic VO.36 [43] to remove adaptors, trim poor quality ends, and delete short sequences (< 36nt). Raw and pre-processed reads were assessed for quality to ensure cleaning efficiency using FastQC (http://www.bioinformatics.babraham.ac.uk/projects/fastqc). Pre-processed reads were assembled using SPAdes Genome Assembler version 3.11.1-Linus [44] using assembly options for paired-end reads and Burrows-Wheeler Aligner mismatch correction. Small (< 500nt) and low (< 2) average kmer coverage contigs were removed from the SPAdes assembly results from further analysis using custom scripts to determine N50, longest contigs, and total length of the contigs.

### Bioinformatic analysis

Virulence-associated genes in each genome were detected using SRST2 [45] against a custom virulence gene database using raw reads. Blastn was used to detect highly divergent virulence-associated genes. Antimicrobial resistance genes in each isolate were detected using SRST2 [45] with default parameters (–min_coverage, 90%, –min_depth 5) by mapping reads from each isolate against the acquired antimicrobial resistance gene database (/data/ARGannot_r3.fasta) provided by SRST2. In addition, the assembled contigs were also blasted against the same antimicrobial resistance gene database, virulence gene database, and candidate hits with identity above 70% and gene coverage above 50% were also reported and merged with the SRST2 results. Serotype was determined by detecting serotype-specific capsule loci [24]. Single nucleotide polymorphisms (SNPs) of *G. parasuis* isolates were identified by running kSNP3 with standard mode. The optimal k-mers size was calculated by the Kchooser program, and the whole-genome phylogeny was analyzed based on identified core genome SNPs [46].

Multilocus sequence typing was performed using SRST2 with an integrated MLST database and definitions. Sequence types (STs) were further confirmed by querying the assembled fasta files using the *G. parasuis* pubMLST typing platform [9, 47]. Assembled fasta files of isolates with novel alleles or allelic profiles were submitted to the PubMLST database. Novel alleles and allelic profiles were extracted from the assembled files and assigned a number using the *G. parasuis* typing database. Novel ST were added to the pubMLST *G. parasuis* database with their corresponding metadata. Determination of clonal complexes was done using the goeburst algorithm [48], a refinement of eBURST [32], implemented in PHYLOViZ (http://www.phyloviz.net), using the stringent six of 7 alleles identical with the founder ST [49]. The global pubMLST *G. parasuis* dataset and the study isolates were subjected to the goeBURST analysis to determine the study isolates' evolutionary descent and clonal complexes. A clonal complex (CC) was defined if it contained at least three STs, including the founder ST. Sequence types that did not meet this criterion were considered singleton STs.

A core-genome single nucleotide polymorphism (SNP) phylogenetic tree was generated by Parsnp V1.2 [50] and visualized by the online tool iTOL [51].

### Data analysis

Data management, descriptive, and inferential analyses of *G. parasuis* serotypes, STs, AMR, and virulence genes were performed in Microsoft Excel® and R (R program version 4.0.0, R core team 2020).

Simpson's index of diversity [52] was used to measure the diversity of genetic type distributions, which included serotype and ST. The calculations followed the formula:

$$\mathrm S\mathrm i\mathrm m\mathrm p\mathrm s\mathrm o\mathrm n'\mathrm s\;\mathrm{index}=1-\lbrack\Sigma\mathrm n\times(\mathrm n-1)\rbrack/\lbrack\mathrm N\times(\mathrm N-1)\rbrack.$$

where N was the total number of isolates, and n was the total number of isolates per serotype or ST.

Tissues of isolation, when reported, were classified as lung, upper respiratory tract (URT), systemic tissues or unknown. Likewise, reported lesions were categorized by affected body system, including nervous, respiratory, systemic, and unknown.Associations between serotype/ST, lineages, *vtaA* genes (1 – 9) versus tissue of isolation, and ST/serotypes versus lineages and *vtaA* genes (1 – 9) were evaluated using logistic regression. To account for the statistical model variation of logistic regression, only serotypes and STs containing at least ten isolates, representing at least 10% of the isolates, were selected for association analyses, which included serotypes 7, 13, 4, 5/12, 2, and NT, and STs 6, 454 and 478. Using Poisson Regression, the total number of Group 1 *VtaA* genes (1—9) was compared across serotypes (or ST).

### Supplementary Information

**Additional file 1.**

**Additional file 2.**

**Additional file 3.**

## Data Availability

The genome assembly data and raw read sequences were deposited in the National Center for Biotechnology Information (NCBI) and Sequence Read Archive (SRA), respectively, under the Bio project number PRJNA749326. Other data is available in the additional files –-.
