# Supplementary material for: Molecular characterization of Glaesserella parasuis strains circulating in North American swine production systems
Source: BMC Vet Res. 2023 Aug 28;19:135. doi: 10.1186/s12917-023-03698-x (PMC10464461; doi:10.1186/s12917-023-03698-x)
Supplement: Supplementary file 1 — Additional file 1. [file 12917_2023_3698_MOESM1_ESM.docx]

Minimum spanning tree (Burst map) showing the relative distribution of the different STs


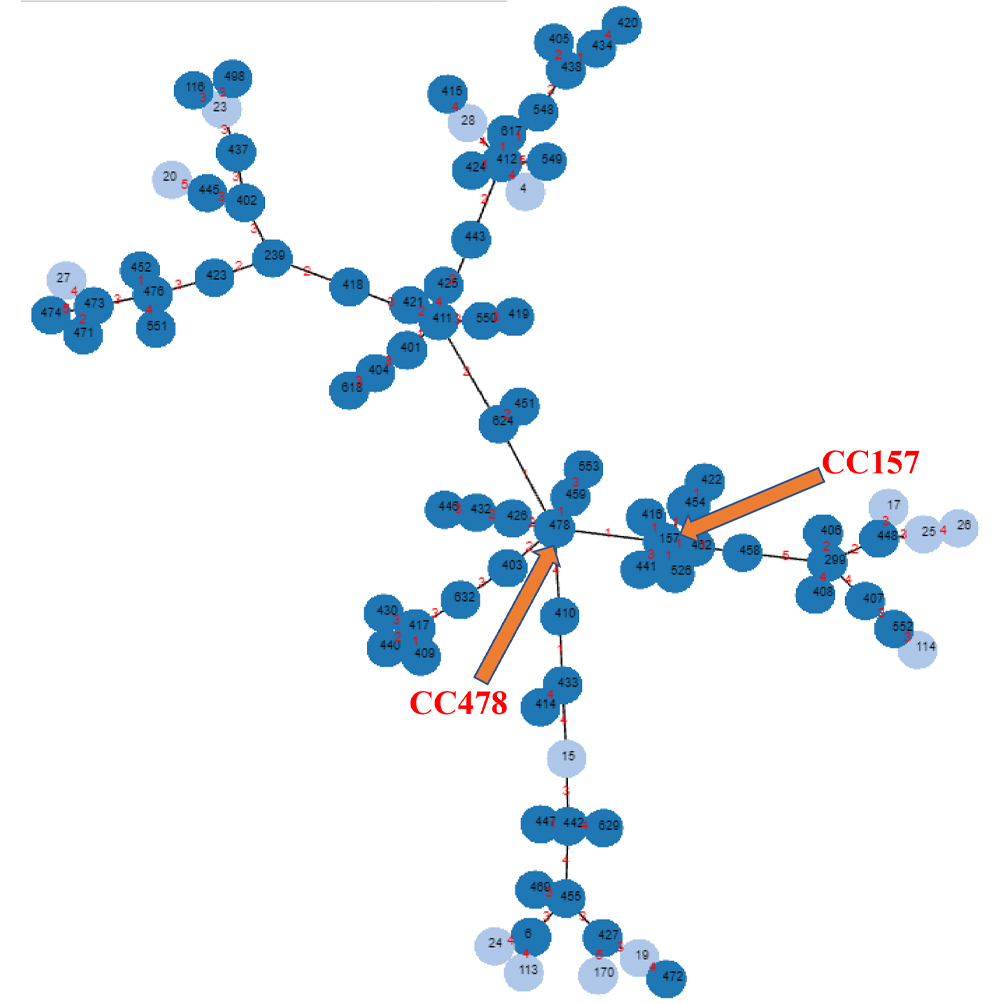


Each circle represents one sequence type. Blue and grey represent STs from field and reference strain isolates. Major clonal complexes, CC157 and CC478 are highlighted.
