# Supplementary material for: Molecular characterization of Glaesserella parasuis strains circulating in North American swine production systems
Source: BMC Vet Res. 2023 Aug 28;19:135. doi: 10.1186/s12917-023-03698-x (PMC10464461; doi:10.1186/s12917-023-03698-x)
Supplement: Supplementary file 2 — Additional file 2. [file 12917_2023_3698_MOESM2_ESM.docx]

Distribution of *G. parasuis* sequence types (ST) and Serotypes within farm flows

| Farm flow | Isolates total | ST (number of isolates) | Serotypes (number of isolates) |
| --- | --- | --- | --- |
| 1 | 1 | 417 (1) | 1 (1) |
| 2 | 1 | 476 (1) | NT (1) |
| 3 | 1 | 459 (1) | 4 (1) |
| 4 | 6 | 454 (6) | 7 (6) |
| 5 | 1 | 454 (1) | 7 (1) |
| 6 | 7 | 408 (1); 452 (1); 454 (5) | 7 (6) |
| 7 | 1 | 462 (1) | 7 (1) |
| 8 | 4 | 454 (4) | 7 (4) |
| 9 | 1 | 157 (1) | 7 (1) |
| 10 | 1 | 420 (1) | 1 (1) |
| 11 | 2 | 418 (1); 454 (1) | 4 (1); 7 (1) |
| 13 | 1 | 454 (1) | 7 (1) |
| 14 | 1 | 424 (1) | 2 (1) |
| 15 | 2 | 430 (2); | 13 (2) |
| 16 | 1 | 415 (1) | 13 (1) |
| 17 | 1 | 455 (1) | 5/12 (1) |
| 18 | 3 | 415 (3) | 13 (3) |
| 19 | 1 | 6 (1) | 5/12 (1) |
| 20 | 1 | 299 (1) | 8 (1) |
| 21 | 4 | 402 (3); 404 (1) | 7 (1); NT (3) |
| 22 | 2 | 415 (1); 454 (1) | 7 (1); 13 (1) |
| 23 | 2 | 476 (2) | 2 (2) |
| 24 | 1 | 414 (1) | 4 (1) |
| 25 | 1 | 404 (1) | NT (1) |
| 26 | 5 | 239 (1); 401(1); 455(1); 474 (2) | 3 (1); 4 (1); 5/12 (3) |
| 27 | 1 | 451 (1) | 2 (1) |
| 28 | 1 | 447 (1) | 7 (1) |
| 29 | 2 | 413 (1); 629 (1) | 2 (1); 4 (1) |
| 30 | 3 | 471 (1); 472 (1); 473 (1) | 4 (1); 7 (2) |
| 31 | 4 | 419 (4); | 13 (4) |
| 32 | 2 | 454 (2) | 7 (2) |
| 33 | 4 | 239 (1); 425 (1); 426 (6) | 1 (1); 2 (2); 4 (1) |
| 34 | 1 | 478 (1) | 7 (1) |
| 35 | 1 | 418 (1) | 4 (1) |
| 36 | 4 | 418 (1); 419 (2); 433 (1) | 4 (2); 13 (2) |
| 37 | 6 | 454 (5); 498 (1) | 1 (1); 7 (5) |
| 38 | 37 | 6 (2); 299 (1); 401 (1); 409 (1); 410 (1); 413 (1); 422 (5); 427 (3); 432 (1); 434 (2); 438 (1); 454 (10); 478 (7); 548 (1) | 2 (9); 4 (2); 5 (4); 6 (5); 7 (9); 8 (1); 13 (4); NT (3) |
| 39 | 1 | 423 (1) | 4 (1) |
| 40 | 1 | 459 (1) | 4 (1) |
| 41 | 17 | 6 (1); 157 (1); 239 (1); 401 (1); 413 (1); 420 (2); 437 (1); 438 (1); 440 (2); 441 (1); 442 (3); 446 (1); 454 (1) | 2 (6); 4 (2); 5/12 (2); 13 (4); 14 (3) |
| 42 | 2 | 443 (1); 452 (1) | 2 (1); 7 (1) |
| 43 | 1 | 116 (1) | 4 (1) |
| 44 | 6 | 6 (6) | 5/12 (3); 13 (3) |
| 45 | 3 | 454 (1); 469 (2) | 7 (1); NT (2) |
| 46 | 3 | 415 (1); 418 (1); 454 (1) | 4 (1); 7 (1); NT (1) |
| 47 | 1 | 454 (1) | 7 (1) |
| 48 | 2 | 239 (1); 433 (1) | 4 (2) |
| 49 | 2 | 421 (2); | 13 (2) |
| 50 | 1 | 458 (1) | 7 (1) |
| 51 | 2 | 411 (1); 459 (1) | 7 (1); 13 (1) |
| 52 | 1 | 412 (1) | 7 (1) |
| 53 | 1 | 478 (1) | 7 (1) |
| 54 | 2 | 416 (2) | 4 (2) |
| 55 | 1 | 6 (1) | 5 (1) |
| 56 | 1 | 458 (1) | 2 (1) |
| 57 | 1 | 548 (1) | 13 (1) |
| 58 | 1 | 448 (1) | NT (1) |
| 59 | 1 | 442 (1) | 7 (1) |
| 60 | 1 | 478 (1) | 7 (1) |
| 61 | 1 | 526 (1) | 7 (1) |
| 62 | 2 | 553 (2) | 13 (2) |
| 63 | 2 | 413 (2) | 2 (2) |
| 64 | 1 | 617 (1) | 7 (1) |
| 65 | 1 | 438 (1) | NT (1) |
